# Prevalence of the *BRCA1 *founder mutation c.5266dupin Brazilian individuals at-risk for the hereditary breast and ovarian cancer syndrome

**DOI:** 10.1186/1897-4287-9-12

**Published:** 2011-12-20

**Authors:** Ingrid P Ewald, Patrícia Izetti, Fernando R Vargas, Miguel AM Moreira, Aline S Moreira, Carlos A Moreira-Filho, Danielle R Cunha, Sara Hamaguchi, Suzi A Camey, Aishameriane Schmidt, Maira Caleffi, Patrícia Koehler-Santos, Roberto Giugliani, Patricia Ashton-Prolla

**Affiliations:** 1 Laboratório de Medicina Genômica, Centro de Pesquisa Experimental - Hospital de Clínicas de Porto Alegre. Rua Ramiro Barcelos 2350. 90035-903. Porto Alegre, RS. Brazil; 2 Programa de Pós-Graduação em Medicina: Ciências Médicas, Universidade Federal do Rio Grande do Sul. Rua Ramiro Barcelos 2400, 2° andar. 90035-903. Porto Alegre, Brazil; 3 Programa de Pós-Graduação em Genética de Biologia Molecular, Universidade Federal do Rio Grande do Sul. Av. Bento Gonçalves, 9500 - Prédio 43323M. 91501-970 - Caixa Postal 15053 - Porto Alegre, RS, Brasil; 4 Departamento de Genética e Biologia Molecular, Centro de Ciências Biológicas e da Saúde, Universidade Federal do Estado do Rio de Janeiro. Rua Frei Caneca, 94. 20211-030 Rio de Janeiro, Brazil; 5 Divisão de Genética, INCA (Instituto Nacional de Câncer). Rua André Cavalcanti, 37 - Centro 20231-050. Rio de Janeiro, Brazil; 6 Laboratório de Genômica Funcional e Bioinformática, Instituto Oswaldo Cruz (IOC-FIOCRUZ), Fundação Oswaldo Cruz, Av. Brasil 4365. Pavilhão Leônidas Deane (Pav. 26) - 1° andar- sala 110 21040-900 - Rio de Janeiro, Brazil; 7 Departamento de Pediatria, Faculdade de Medicina da Universidade de São Paulo. Av. Dr. Enéas Carvalho de Aguiar 647. 05403-900, São Paulo, Brazil; 8 Instituto Israelita de Ensino e Pesquisa Albert Einstein. Av. Albert Einstein 627, 05651-901. São Paulo, Brazil; 9 Instituto de Matemática, Universidade Federal do Rio Grande do Sul. Av. Bento Gonçalves 9500 Prédio 43-111 - Agronomia. Caixa Postal 15080, Porto Alegre, Brazil; 10 Núcleo Mama Moinhos. Associação Hospitalar Moinhos de Vento. Rua Ramiro Barcelos 910, 11°. Andar. 90035-001, Porto Alegre, Brazil; 11 Serviço de Genética Médica, Hospital de Clínicas de Porto Alegre. Rua Ramiro Barcelos 2350. 90035-903. Porto Alegre, RS. Brazil; 12 Departamento de Genética, Universidade Federal do Rio Grande do Sul. Av. Bento Gonçalves, 9500 - Prédio 43323. Caixa Postal 15053 - 91501-970. Porto Alegre, Brazil; 13 Instituto Nacional de Genética Médica Populacional - INAGEMP. Hospital de Clínicas de Porto Alegre. Rua Ramiro Barcelos 2350. 90035-903. Porto Alegre, RS. Brazil

**Keywords:** Hereditary breast cancer, Hereditary breast and ovarian cancer Syndrome, Founder mutations, *BRCA1 *gene, *BRCA2 *gene

## Abstract

About 5-10% of breast and ovarian carcinomas are hereditary and most of these result from germline mutations in the *BRCA1 *and *BRCA2 *genes. In women of Ashkenazi Jewish ascendance, up to 30% of breast and ovarian carcinomas may be attributable to mutations in these genes, where 3 founder mutations, c.68_69del (185delAG) and c.5266dup (5382insC) in *BRCA1 *and c.5946del (6174delT) in *BRCA2*, are commonly encountered. It has been suggested by some authors that screening for founder mutations should be undertaken in all Brazilian women with breast cancer. Thus, the goal of this study was to determine the prevalence of three founder mutations, commonly identified in Ashkenazi individuals in a sample of non-Ashkenazi cancer-affected Brazilian women with clearly defined risk factors for hereditary breast and ovarian cancer (HBOC) syndrome. Among 137 unrelated Brazilian women from HBOC families, the *BRCA1*c.5266dup mutation was identified in seven individuals (5%). This prevalence is similar to that encountered in non-Ashkenazi HBOC families in other populations. However, among patients with bilateral breast cancer, the frequency of c.5266dup was significantly higher when compared to patients with unilateral breast tumors (12.1% vs 1.2%, *p *= 0.023). The *BRCA1 *c.68_69del and *BRCA2 *c.5946del mutations did not occur in this sample. We conclude that screening non-Ashkenazi breast cancer-affected women from the ethnically heterogeneous Brazilian populations for the *BRCA1 *c.68_69del and *BRCA2 *c.5946del is not justified, and that screening for *BRCA1*c.5266dup should be considered in high risk patients, given its prevalence as a single mutation. In high-risk patients, a negative screening result should always be followed by comprehensive *BRCA *gene testing. The finding of a significantly higher frequency of *BRCA1 *c.5266dup in women with bilateral breast cancer, as well as existence of other as yet unidentified founder mutations in this population, should be further assessed in a larger well characterized high-risk cohort.

## Introduction

Breast cancer is the most common non-cutaneous malignancy in Brazilian women of all ages. In the Southern and Southeastern States of Brazil, the estimated breast cancer incidence rates for 2010 reached 64.54 and 64.30 per 100,000 women, the highest in the country [1]. In spite of continuous efforts to improve early detection and treatment, breast cancer remains the leading cause of deaths by cancer in Brazilian women. Furthermore, mortality rates by this type of cancer are still increasing in the Southern States of the country [2,3].

It is estimated that 5-10% of breast cancers are hereditary, arising from highly penetrant germline mutations in cancer predisposition genes [4]. A significant proportion of individuals with hereditary breast cancer have mutations in the tumor suppressor genes *BRCA1 *(OMIM # 113705) and *BRCA2 *(OMIM # 600185) [5]. Carriers of such mutations are usually predisposed to breast, ovarian, prostate and other cancers at an early age, a syndrome known as Hereditary Breast and Ovarian Cancer (HBOC) [6].

*BRCA1 *and *BRCA2 *are similar in their structure and quite large (100 and 70 kb, respectively). Germline mutations in these genes are usually point mutations, and are scattered along their entire coding sequences; mutational hot-spots are uncommon. Deleterious gene rearrangements may also occur in up to 30% of the cases [7]. Therefore, full gene sequencing and rearrangement testing is warranted for accurate diagnosis in most individuals. In a few populations, however, founder mutations have been described and are responsible for a significant proportion of the mutation-positive diagnoses. This is the case for the c.68_69del and c.5266dup mutations in *BRCA1 *and c.5946del mutation in *BRCA2 *(until recently referred in the literature as 185delAG, 5382insC and 6174delT, respectively) which are found in 10-12% of Ashkenazi Jewish women diagnosed with breast cancer [8,9]. *BRCA *gene founder mutations have also been described in other populations, including the Slavic (*BRCA1*c.5266dup mutation) [10,11], Finnish and Icelandic (*BRCA2*999del5 mutation) and German populations (*BRCA1*delexon17) [12,13]. In these populations, initial screening of a clinically suspicious case by testing for founder mutations is acceptable and allows the diagnosis of a significant number of carriers using fast and straightforward methodologies at a lower cost [14].

In a recent study done in the Brazilian State of Rio de Janeiro [15], 402 unrelated non-Ashkenazi women affected with breast cancer were screened for the three Ashkenazi founder mutations. Of the nine mutation-positive individuals identified, five (56%) harbored the *BRCA1*c.5266dup mutation in exon 20 and the overall prevalence of this particular mutation in the sample studied was 1.24%. The women enrolled in the study were not selected for a family history of the disease, and their ancestry was not described. A previous study [16] of 47 unrelated breast cancer-affected women from Rio de Janeiro with a family history of cancer suggestive of the HBOC syndrome had already identified the *BRCA1*c.5266dup mutation in a significant proportion of the mutation-positive patients (four in seven). Interestingly, none of them reported Jewish ancestry. Finally, Simon *et al*. [17] reported the occurrence of the *BRCA1*c.5266dup mutation in Jewish and non-Jewish HBOC families from the Brazilian State of São Paulo. Recently, Hamel *et al*. [18] have studied the c.5266dupC in 14 different population groups (Russian, Latvian, Ukrainian, Czech, Slovak, Polish, Danish, Dutch, French, German, Italian, Greek, Brazilian and AJ) and confirmed that all carriers of this mutation share a common haplotype proposing that it arose approximately 1800 years ago in either Scandinavia or northern Russia and subsequently spread to the various populations, including the Ashkenazi Jewish population.

Considering the importance of identifying mutation-positive HBOC patients for genetic counseling purposes and the previous reports indicating that founder mutations, in particular *BRCA1*c.5266dup, may be common in Brazilian patients diagnosed with breast cancer, we performed this study with the aim of determining the frequency of these mutations in non-Ashkenazi individuals diagnosed with cancer and whose families fulfilled clinical criteria for the HBOC syndrome.

## Patients and Methods

A consecutive sample of 137 unrelated Brazilian patients, self-referred as non-Ashkenazi and with a significant personal and/or family history of hereditary breast and ovarian cancer, was evaluated at cancer genetic counseling services from the two regions with the highest breast cancer incidence rates in the country in Southern and Southeastern Brazil: Porto Alegre (from the Cancer Genetics Clinic of Hospital de Clínicas de Porto Alegre) and Rio de Janeiro, (from INCA, the Brazilian National Cancer Institute). All patients confirmed residence in the States from which they were recruited for at least ten years, had been diagnosed with cancer or were cancer unaffected individuals likely to be obligate carriers of *BRCA *mutations. A significant family history of HBOC was defined as presence of either: (a) the American Society of Clinical Oncology (ASCO) criteria for HBOC [19] or (b) a prior probability of harboring a *BRCA *mutation ≥ 30% by pedigree analysis using the Myriad mutation prevalence tables or the Penn II mutation prediction model [20-22]. Women diagnosed with bilateral breast cancer under the age of 50 years, regardless of family history, were also included. All patients relied exclusively on the public health care system through which *BRCA1 *and *BRCA2 *full gene sequencing is currently not available, even for patients of very high genetic risk. After signature of informed consent, genomic DNA was extracted from peripheral blood by conventional methods [23] and screening for the founder mutations was performed by PCR-amplification and DNA sequencing in both directions using the DYEnamic ET Terminator Cycle Sequencing Kit and the MegabaseorABI automated sequencers. The amplification and sequencing primer sequences used were: (a) for *BRCA1 *exon 2 (c.68_69del mutation): 5'-GTTCTTTGGTTTGTATTATTCT-3' and 5'-AGAGGCAGAGTGGATGGA-3'; (b) for *BRCA1 *exon 20 (c.5266dup mutation): 5'-ATATGACGTGTCTGCTCCAC-3' and 5'-GGGAATCCAAATTACACAGC-3'. and (c) for *BRCA2*exon 11 (c.5946del mutation): 5'-AACGAAAATTATGGCAGGTTGTTAC-3' and 5'-GCTTTCCACTTGCTGTACTAAATCC-3'. All mutation-positive samples were confirmed in a second independent analysis. The amplification and sequencing protocols are available upon request.

### Statistical Analyses

Sample size for this study was estimated using WINPEPI (PEPI-for-Windows). SPSS version 18.0 was used for data handling and statistical analyses. For descriptive analysis, categorical variables were described by their absolute and/or relative frequencies and quantitative variables were expressed as mean and standard deviation (SD). The existence of an association between categorical variables was examined by chi-square. Association between bilateral breast cancer and the c.5266dup mutation in a subgroup analysis of patients with breast cancer were analyzed by Fishers' exact test. A significance level of 0.05 was adopted.

## Results

Sample characteristics are described in Table 1. The mean age at cancer diagnosis (all types) was 43.4 years (SD = 10.1; range: 24-73 years). Among the 137 probands included in the study, 42 (31.0%) were recruited in Porto Alegre and 95 (69.0%) in Rio de Janeiro.

**Table 1:** Clinical description of the sample studied (n = 137)

Characteristic	N	%	Mean (SD)
**Sex**			
Female	134	97.8	
Male	3	2.2	
Age at breast cancer (years)*			42.3 (8.3) range: 24-73
**Inclusion criteria**			
ASCO	113	82.5	
Probability of mutation (Myriad) ≥ 30%	62	45.3	
Probability of mutation (PENN II model) ≥ 30%	28	20.4	
Bilateral breast cancer	34	24.8	
Cancer history in the proband (n = 136)**			
One primary tumor			
Breast cancer	85	62.5	
Ovarian cancer	4	2.9	
Other#	5	3.7	
Multiple primaries:			
≥ 2 Breast	31	22.8	
≥ 2 ovarian	0	0	
1breast and 1 ovarian	2	1.5	
≥ 2 Breast and 1 ovarian	3	2.2	
At least one breast + other	5	3.7	
At least one ovarian + other	1	0.7	
**Criteria for multiple hereditary cancer syndromes**			
HBOC + Li-FraumeniLike (Eeles criteria)	23	16.8	
HBOC + Li-Fraumeni Like (Birch criteria)	2	1.4	
HBOC + Hereditary Breast and Colorectal Cancer	14	10.2	
HBOC + Hereditary Non-Polyposis Colorectal Cancer	3	2.2	
**Cancer family history (1^st ^and 2^nd ^degree relatives)**			
Number of breast cancer cases			3.3 (1.52)
Number of ovarian cancer cases			1.3 (0.61)
Age at first breast cancer			44.2 (6.8)

*In women with multiple breast cancers, age at the diagnosis of the first primary was considered.

**One patient was cancer unaffected. In the sample of 136 cancer affected probands, there were a total of 184 tumors, including synchronous and metachronous cases.

^#^(colorectal, melanoma, gastric, uterine cervix, esophagus, endometrium)

Most patients (n = 126, 91.9%) had been diagnosed with breast cancer and of these, 110 (80.3%) were diagnosed under the age of 50 years. Five patients had been diagnosed with tumors other than breast and ovarian cancer (colorectal cancer, melanoma, gastric cancer, esophageal cancer, endometrial cancer), but were included because they had a significant family history of breast and ovarian cancer in first and second degree relatives. The only cancer-unaffected patient in this study was considered an obligate carrier due to a significant history of breast cancer in the parental and offspring generations.

Approximately 30.7% of the patients had synchronous or metachronous multiple primary tumors (*n *= 42) and the mean age at first cancer diagnosis in this group was 41.5 years (SD = 6.9; range: 28-61 years). Approximately one-fourth of the total participants had bilateral breast cancer (n = 34), and the mean age at the first primary tumor in this group was 42.2 years (SD = 7.2; range 28-61 years), similar to the mean age in patients with only one primary breast cancer (42.3 years; SD = 8.8; range 24-73).

For the entire sample, 45.3% and 20.4% had an estimated probability of a *BRCA *gene mutation equal or greater than 30.0% using the Myriad prevalence tables and the Penn II model, respectively. The mean prior probability of carrying a *BRCA *mutation using either method was similar: 24.4% using the Myriad tables (SD = 3.4, range, 2.8-67.2%) and 22.9% using Penn II (SD = 15.1, range, 7-93%).

Overall, a germline mutation was identified in seven (5.0%) of the 137 patients studied and in all of them, it consisted of the c.5266dup mutation in exon 20 of the *BRCA1 *gene. A more detailed description of the mutation-positive cases is shown in Table 2. The other founder mutations (c.68_69del in *BRCA1 *and c.5946del in *BRCA2*) were not identified. If only breast or ovarian cancer-affected probands are considered, the *BRCA1*c.5266dup frequency remains similar, 5.3% (7/131).

**Table 2:** Detailed description of *BRCA1*c.5266dup-positive probands and their families

						Prior Probability of Mutation in a *BRCA *gene	
Case #	Cancer diagnosis (index-case)	Age at diagnosis (ys)	Cancer family history*	ASCO criteria	Limited Family Structure	Myriad Prevalence Tables (%)	Couch Prediction Model (%)
**1-RJ**	Breast	33	MAT Br (F-45)	Yes	No	16.3	20.0
**2-RJ**	Ovarian	47	PAT Ov (60), Br (F-30), End (64)	Yes	No	46.8	44.0
**3-RS**	Ovarian	52	MAT Br (F-44), Ov (F-76), Ov (F-66), Ut (F-78), Ut (N/A), Ga (M-68), CRC (M-69), HeN (M-N/A), HeN (M-75)	No	No	46.8	43.0
			PAT Lu (M-N/A), CRC (M-N/A), CRC (F-N/A), Ut (35), Br (M-62), Ga (M-N/A), Br (F-36), Bilat Br (F-45), Br (F-44), Br (F-45)	Yes	No	40.8	33.0
**4-RJ**	Bilateral Breast	45 and 50	MAT BilatBr (F-45,50), Ov (39) Br (F-49), Bilat Br (F-47,50)	Yes	No	40.7	31.0
**5-RJ**	Bilateral Breast	46 and 47	PAT Lu (M-N/A), Ga (M-N/A) CRC (M-N/A)	No	Yes	6.9	9.0
**6-RJ**	Bilateral Breast	33 and 38	-	No	Yes	6.9	15.0
**7-RS**	Bilateral Breast	35 and 45	MAT Ov (F-58), Ov (F-49), Br (F-90) Br (F-49), Ga (M-70), Liv (M-70)	Yes	No	40.7	47.0

Legend: RJ: family recruited from Rio de Janeiro; RS = family recruited from Porto Alegre;

(*) MAT = cancer history in maternal side of the family, PAT = cancer history in paternal side of the family; other cancer diagnoses in family are indicated by the abbreviated cancer type (Br = breast, Lu = lung; Ga = gastric Ov = ovarian; Prost = prostate; Esoph = esophageal; Liv = liver; End = endometrial; CRC = Colorectal; HeN = head and neck cancer; Ut = uterine cancer, not defined whether cervix or endometrium) followed by followed by sex (M = male, F = female) and age at diagnosis (N/A = not available).

Patients diagnosed with bilateral breast cancer were more likely to carry the *BRCA1*c.5266dup mutation than patients with unilateral breast cancer (*BRCA1*c.5266dup was present in 4 of 34 patients with bilateral breast cancer vs. 1 of 82 unilateral breast cancer patients; *p *= 0.023). In addition, *BRCA1*c.5266dup carriers with bilateral breast cancer were younger when first diagnosed (mean age 38 years; SD = 6; range 33-47) than non-carriers (43 years; SD = 7; range 28-61) although this difference did not reach significance (*p *= 0.189).

Two of the four metachronous breast cancer mutation carriers had no family history of breast or ovarian cancer and did not fulfill any other inclusion criteria of the study, apart from the early onset bilateral breast cancer diagnosis. Both cases, however, had a limited family structure (fewer than 2 first- or second-degree female relatives alive and at an age ≥ 45 years in either lineage) as defined by Weitzel *et al*. [24].

Families from the Southern region had a significantly higher number of breast cancer diagnoses in the family than those from the Southeast (4.0 in versus 3.1, *p *= 0.004). However, there was no significant difference in the mean age of breast cancer diagnosis (40.4 and 42.8 years, respectively, *p *= 0.206), nor in *BRCA1*c.5266dup frequency (3.8% and 4.5%, respectively, p > 0.999) between regions.

## Discussion

The identification of individuals at-risk for hereditary breast cancer is important to ensure that appropriate risk reducing interventions are offered, to counsel patients and families regarding recurrence risk and to guide the decisions about cancer treatment interventions in affected individuals. The precise identification of at-risk individuals in a given family depends on the identification of a deleterious germline mutation in a cancer predisposition gene. In the case of the HBOC syndrome, genetic testing is often hampered by the complexity and cost of testing the *BRCA *genes, especially in lower resource countries. In Brazil, such testing is not yet covered by private insurance nor provided by the public health care system and its cost in private laboratories precludes its use for most at-risk families.

The initial screening of founder mutations in *BRCA1 *and *BRCA2 *before investigation of their entire coding regions has been well established in individuals of Ashkenazi Jewish ancestry as well as in a few other populations, and it is considered a cost-effective approach in these populations [25,26]. In other Latin American communities, this issue has not been largely explored. An exception is a large cohort of Latin American HBOC families studied by Weitzel et al. (2005) [27] in which six recurrent mutations in *BRCA1 *accounted for 47% of the deleterious germline mutations and interestingly, the *BRCA1 *c.68_69del mutation, one of the Ashkenazi Jewish founder mutations, occurred in 3.6% of this clinic-based cohort of predominantly Mexican women. In this study the authors suggest that an initial diagnostic screen with a panel for these more commonly observed mutations could be cost-effective.

Only two reports from Brazilian breast-cancer affected patients have suggested that a founder *BRCA1 *mutation, c.5266dup, may be encountered at a significant prevalence [16,17]. This mutation is the second most common mutation described in the Breast Cancer Information Core (BIC) database [28] for HBOC families worldwide. It has been reported in 14.0%, 10.0%, 6.0% and 4.0% of Ashkenazi Jewish, German, Italian and Russian women with breast cancer [29-32], respectively. It was also described in a recent study from Portugal, where it appeared in approximately 1.0% of the series studied [33]. In addition, in a recent multi-national study, Hamel *et al*. (2011) [18] identified this mutation in several European countries. To our knowledge, it has not been detected in Spain [34,35] nor in other South American countries, although only a few comprehensive mutation studies (i.e. including sequencing of the entire coding region of both genes and gene rearrangement testing) in HBOC families have been produced in these regions [34-40]. The penetrance of the *BRCA1*c.5266dup mutation has been well defined in Ashkenazi women, being associated with a cumulative lifetime risk of 0.67 for breast cancer and 0.33 for ovarian cancer [41].

In the present study, we screened 137 unrelated and self-referred non-Ashkenazi women at high risk for the HBOC syndrome for the three common founder mutations described in Ashkenazi Jewish cohorts and only *BRCA1*c.5266dup was identified, at a frequency of 5.0%. As expected, this frequency is higher than that described by the other Brazilian studies, including that of Gomes *et al*. [15] and Esteves et al [42], and likely results from the study design, that defined a significant personal and familial cancer history as inclusion criterion at recruitment.

Our results are in agreement with previous prevalence studies of the c.5266dup mutation in other high-risk non-Ashkenazi populations, such as the Italian and German populations [30,31]. The origin of the *BRCA1*c.5266dup mutation in Brazilian patients and its relation to the Eastern-European counterpart remain to be determined. In a recently published study, da Costa *et al*. (2008) [43] evaluated the haplotypic profile of seven Brazilian carriers of c.5266dup and reported that all mutation carriers shared an identical haplotype, indicating a common origin. Some authors (i.e. Carvalho Silva *et al*. [44] and Gomes *et al*. [15]) have postulated that the entry of this mutation into Brazil is related to immigration of European Jews from Portugal in the sixteenth century. In the report by Weitzel *et al*. (2005) [27], that identified the Ashkenazi founder mutation c.68_69del in Mexican HBOC families, all mutation carriers shared the Ashkenazi Jewish founder haplotype. The authors postulate that Mexican carriers of these mutations are likely descendants of *Conversos *who immigrated to the Americas in the 15^th^-16^th ^centuries and over generations assimilated into the larger Hispanic society. The same reasoning may be applied to the occurrence of *BRCA1*c.5266dup in Brazil. However, the absence of this mutation in studies from Spain or from other South American countries and the fact that only one case was reported in Portugal is against this hypothesis. Recent results from the study of Hamel *et al*. (2011) [18], show that the mutation probably originated in Scandinavia or Northern Russia, was then disseminated in European populations and thus could have entered Brazil through one or more of the several large European immigration waves in the 18^th ^and 19^th ^centuries. A detailed understanding of its entrance and distribution in Brazil, remains to be determined [45,46].

One interesting finding of our study is the high frequency of the c.5266dup mutation in patients with bilateral breast cancer, compared to those with unilateral breast cancer. It is known that women with bilateral breast cancer are more likely to carry a *BRCA *mutation [47,48]. Gershoni-Baruch *et al*. (1999) [49], studying Jewish women with bilateral breast cancer, found a high prevalence of founder mutations (31.0%), and 3.7% of the cases carried the c.5266dup mutation. In their report, bilateral breast cancer *per se *did not seem to reflect genetic predisposition unless associated with early age of onset. In another study that compared mutation prevalence in women with unilateral, family history positive and early onset breast cancer and women with bilateral breast cancer, the distribution of *BRCA1*c.5266dup was not significantly different between groups [32].

A limitation of our study is that we have not investigated the potential existence of other founder mutations that could become apparent after gene full gene sequencing; however, there is currently no published evidence for Brazilian *BRCA *founder mutations other than c.5266dup. We did not consider testing for founders described in Hispanic populations (i.e. Weitzel *et al*. (2005) [27]; Rodriguez *et al*. (2008) [50]) since it is our understanding that the population from the Brazilian cities studied here show trihybrid ancestries that are distinct from the Central American populations as indicated by studies from Alves-Silva *et al*. (2000) [51] and Parra *et al*. (2003) [52]. Additionally, it has been demonstrated clearly that even *mestizos *from different Central and South American regions have significant intra- and interethnic variability and admixture profiles [53].

Finally, a debatable point regarding founder mutation frequency in a given population or high risk group, is how much is enough to justify screening. Although the economic analysis of such a question is beyond the scope of our study, there must be also a concern with the social and medical consequences of such testing. Given current knowledge, if a recommendation for screening of founder *BRCA *mutations is made for Brazilian HBOC women without subsequent comprehensive testing, most (likely > 90%) of the mutations carriers would remain unidentified. Such a recommendation could be erroneously interpreted as sufficient, or result in false tranquilization whenever negative, hampering continuation towards mutation testing of the entire coding region of both genes, which could obviously have serious consequences for these individuals and their families. This concern is further justified by the inaccessibility to proper cancer genetic counseling for most high-risk Brazilian women, and by the lack of coverage through both private and public health-based insurance of germline mutation testing. Thus, although the frequency of one single mutation, *BRCA1*c.5266dup is significant in this subset of Brazilian women with a personal and familial history of HBOC, initial screening for one founder mutation in at-risk patients should be performed in the setting of genetic counseling and within a cancer risk evaluation program. In a high-risk patient or family, testing should always include a comprehensive approach (full gene sequencing and rearrangement testing for both *BRCA *genes) when the initial founder mutation screening is negative.

We conclude that initial screening of the *BRCA1 *c.5266dup mutation in Brazilian individuals at high risk for HBOC is justified, but should be considered with caution. This statement is based on the frequency of the single founder mutation currently described in this population, lack of evidence of other founder *BRCA *mutants at a significant frequency in Brazil, and potential consequences of misinterpretation of negative screening results. These principles may apply to other highly admixed populations in South America and other continents.

It is imperative to concentrate efforts in making *BRCA *mutation testing and genetic counseling available to all high-risk patients in Brazil, and in educating health care professionals in the identification and proper management of these individuals. The best strategy for mutation screening and molecular diagnosis of high risk HBOC families in Brazil will only be fully understood after a comprehensive knowledge of *BRCA *mutation types and frequencies in this population.

Finally, the fact that two of the seven mutation-positive individuals had a limited family structure reinforce the importance of recognizing this feature and demonstrate that criteria for genetic testing must be adapted to consider this variable. The increased frequency of *BRCA1*c.5266dup in women with bilateral breast cancer should be further assessed in a larger cohort.

## Abbreviations

ASCO: American Society of Clinical Oncology; BIC: Breast Cancer Information Core; *BRCA1*: Breast Cancer 1; *BRCA2*: Breast Cancer 2; CAPES: Coordenação de Aperfeiçoamento de Pessoal de Nível Superior; CNPq: Conselho Nacional de Desenvolvimento Científico e Tecnológico; FIPE - HCPA: Fundação de Incentivo à Pesquisa do Hospital de Clínicas de Porto Alegre; FAPERGS: Fundação de Amparo à Pesquisa no Rio Grande do Sul; HBOC: Hereditary Breast and Ovarian Cancer; PCR: Polymerase Chain Reaction; PRODOC: Programa de Apoio a Projetos Institucionais com a Participação de Recém - Doutores.

## Competing interests

The authors declare that they have no competing interests.

## Authors' contributions

IPE carried out the molecular genetic studies, sequencing analysis and drafted the manuscript. PI participated in data collection, data analysis and drafted the manuscript. FRV, MAMM and ASM participated in patient recruitment, data collection and revision of the manuscript. CAMF, DRC and SH participated in sequencing analysis. SAC contributed to study design, critical data analysis and coordinated statistical analyses. AS participated in statistical analyses. MC contributed to study design and participated in patient recruitment. PKS participated in sequencing analysis and critical data analysis. RG participated in supervision of IPE, drafting and revision of the manuscript. PAP conceived of the study and supervised data collection, molecular genetic studies, data analysis and manuscript revision. All authors read and approved the final manuscript.
